# Crystal structure of *catena*-poly[[(dimethyl sulfoxide-κ*O*)(pyridine-2,6-di­carboxyl­ato-κ^3^ *O*,*N*,*O*′)nickel(II)]-μ-pyrazine-κ^2^ *N*:*N*′]

**DOI:** 10.1107/S2056989016007064

**Published:** 2016-04-29

**Authors:** Chen Liu, Annaliese E. Thuijs, Ashley C. Felts, Hamza F. Ballouk, Khalil A. Abboud

**Affiliations:** a Department of Chemistry and Environmental Science, Grenfell Campus, Memorial University of Newfoundland, Corner Brook, NL, A2H 5G4, Canada; b Department of Chemistry, University of Florida, Gainesville, FL 32611-7200, USA

**Keywords:** crystal structure, one-dimensional Ni^II^ coordination polymer, pyridine-2,6-di­carb­oxy­lic acid, pyrazine, π–π stacking, C—H⋯ π inter­action, hydrogen bonding

## Abstract

The title coordination polymer, [Ni(C_7_H_3_NO_4_)(C_4_H_4_N_2_)(C_2_H_6_OS)]_*n*_, consists of [010] chains composed of Ni^II^ ions linked by bis-monodentate-bridging pyrazine mol­ecules. Each of the two crystallographically distinct Ni^II^ ions is located on a mirror plane and is additionally coordinated by a dimethyl sulfoxide (DMSO) ligand through the oxygen atom and by a tridentate 2,6-pyridine-di­carb­oxy­lic acid dianion through one of each of the carboxyl­ate oxygen atoms and the pyridine nitro­gen atom, leading to a distorted octa­hedral coordination environment. The title structure exhibits an inter­esting complementarity between coordinative bonding and π–π stacking where the Ni—Ni distance of 7.0296 (4) Å across bridging pyrazine ligands allows the pyridine moieties on two adjacent chains to inter­digitate at halfway of the Ni—Ni distance, resulting in π–π stacking between pyridine moieties with a centroid-to-plane distance of 3.5148 (2) Å. The double-chain thus formed also exhibits C—H⋯π inter­actions between pyridine C—H groups on one chain and pyrazine mol­ecules on the other chain. As a result, the inter­ior of the double-chain structure is dominated by π–π stacking and C—H⋯ π inter­actions, while the space between the double-chains is occupied by a C—H⋯O hydrogen-bonding network involving DMSO ligands and carboxyl­ate groups located on the exterior of the double-chains. This separation of dissimilar inter­actions in the inter­ior and exterior of the double-chains further stabilizes the crystal structure.

## Abstract 2

A one-dimensional Ni^II^ coordination polymer has been prepared *via* solvothermal synthesis using dimethyl sulfoxide as solvent. The coordination polymer forms double-chains along [010] and exhibits π–π stacking and C—H⋯π inter­actions forming the inter­ior of the double-chains, separated from a C—H⋯π hydrogen-bonding network in the space between the double-chains.

## Chemical context   

In general, π–π inter­actions are considered important mechanisms for mol­ecular recognition and may function as structure-directing factors in the design and preparation of coordination polymers. However, π–π inter­actions are not always observed in the final coordination polymer simply by using starting materials containing aromatic moieties. During our investigation of the rational design and synthesis of coordination polymers, we have previously reported a dinuclear Ni^II^ complex obtained by reacting 2,6-pyridine di­carb­oxy­lic acid and nickel carbonate using water as solvent (Liu *et al.*, 2011 ▸). The inter­molecular force between the dinuclear complexes is dominated by hydrogen bonding. We recently repeated the synthesis of this compound using dimethyl sulfoxide (DMSO) as solvent under solvothermal conditions and obtained the title compound. We herein report its synthesis and structure which exhibits both π–π stacking and C—H⋯π inter­actions involving two different aromatic mol­ecules, *viz*. pyridine and pyrazine.
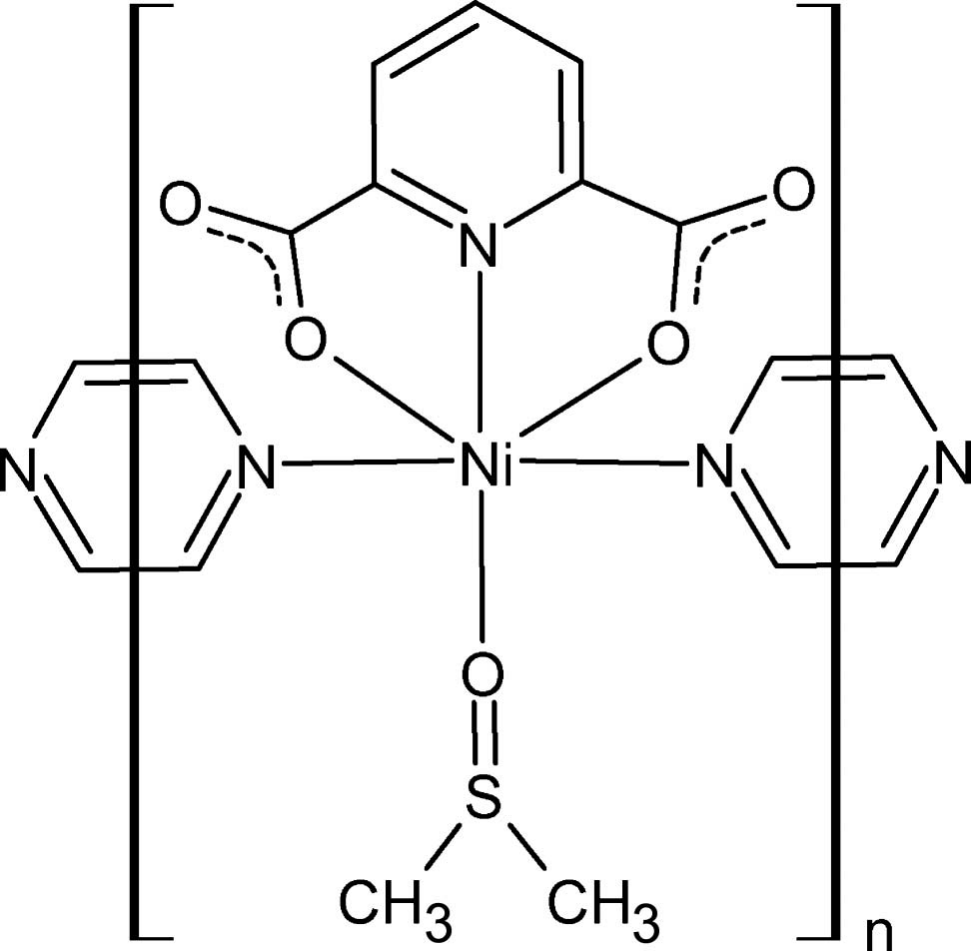


## Structural commentary   

**Figure 1:**
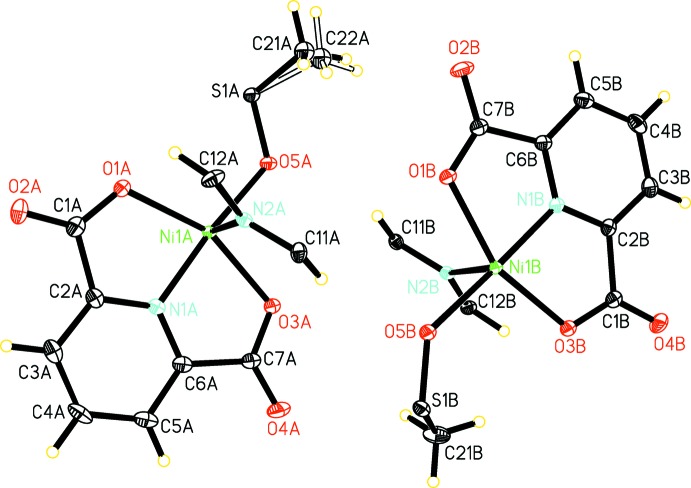
A view of the asymmetric unit of the title compound, showing the atom labelling. Displacement ellipsoids are drawn at the 50% probability level. All disordered components are shown.

The asymmetric unit contains two half Ni^II^ complexes with mirror symmetry (denoted *A* and *B*), where each of the Ni^II^ atoms is coordinated by a 2,6-pyridine-di­carb­oxy­lic acid dianion, a pyrazine mol­ecule, and a DMSO ligand (Fig. 1 ▸). The tridentate 2,6-pyridine-di­carboxyl­ate anion coordinates to Ni^II^ in a meridional fashion *via* the pyridine nitro­gen atom and two carboxyl­ate oxygen atoms; the DMSO mol­ecule coordinates to Ni^II^ through its oxygen atom and the pyrazine ligands through their N atoms. Thus each Ni^II^ is in an N_3_O_3_ coordin­ation environment. Individual Ni^II^ complexes are linked along the axial positions by bis-monodentate bridging pyrazine mol­ecules to form a linear chain parallel to [010] and propagated through mirror symmetry elements passing through the Ni^II^ atoms, the anions, and bis­ecting both the pyrazine ligands and the DMSO mol­ecules along the S=O bonds. In the chains, the Ni—Ni distance across bridging pyrazine is 7.0296 (4) Å, *i.e.* the length of the *b* axis.

## Supra­molecular features   

**Figure 2:**
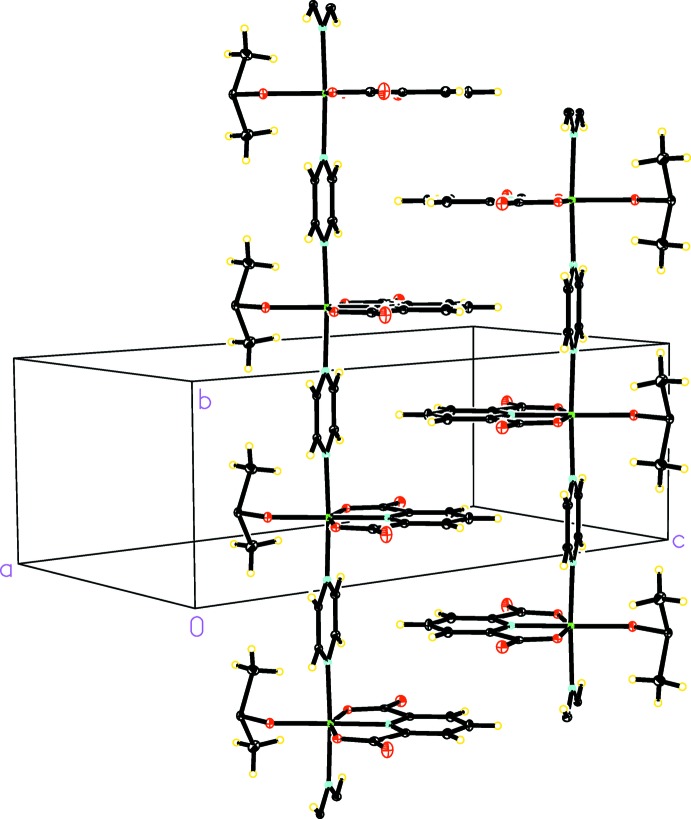
A view of the double-chain structure of the title compound running parallel to [010].

In the crystal, two Ni^II^ chains form a double-chain structure *via* π–π stacking between their pyridine moieties (Fig. 2 ▸). Two stacked pyridine rings in the double-chain structure are separated by a centroid-to-plane distance of 3.5148 (2) Å. This separation distance is half of the Ni—Ni distance, indicating that the formation of π–π stacking in the double-chain structure may have been promoted by coordinative bonding distances across bridging pyrazine ligands. A search in the literature returned only a few other examples of coordination polymers exhibiting similar structural features (Zheng *et al.*, 2000 ▸; Nawrot *et al.*, 2015 ▸). Within the double-chain, two π–π stacked pyridine moieties are also parallel-shifted by 1.50422 (8) Å, consistent with values obtained from computational studies (Huber *et al.*, 2014 ▸). Although π–π stacking inter­actions are prevalent among systems composed of discrete aromatic mol­ecules, it is not always observed in coordination polymers synthesized from aromatic starting materials. The title structure thus provides an inter­esting example for further investigation on the inter­play between coordinative bonding and π–π stacking as a potential strategy for incorporating π–π stacking in the design and synthesis of coordination polymers.

Accompanying the π–π stacking inter­action described above, there is also a T-shaped C—H⋯π inter­action between the pyridine C4—H4 group and the bridging pyrazine mol­ecule (Tiekink & Zuckerman-Schpector, 2012 ▸), contributing additional stability to the double-chain structure. The concurrence of both parallel π–π stacking and T-shaped C—H⋯π inter­actions in crystal structures is known in the literature, but primarily among systems of discrete aromatic mol­ecules (Tiekink & Zuckerman-Schpector, 2012 ▸). We are aware of only one other example of a coordination polymer exhibiting this feature (Felloni *et al.*, 2010 ▸). In the C —H⋯π configuration of the title structure, the centroid-to-centroid distance between pyridine and pyrazine is 4.8389 (2) Å, which includes the pyridine C4—H4 bond length of 0.95 Å and a distance of 2.53310 (12) Å from the pyridine H4 atom to the centroid of the pyrazine ring. Although the title structure is a coordination polymer, these distances are in good agreement with results of computational studies performed on discrete aromatic mol­ecules (Mishra & Sathyamurthy, 2005 ▸; Hohenstein & Sherrill, 2009 ▸; Huber *et al.*, 2014 ▸).

**Figure 3:**
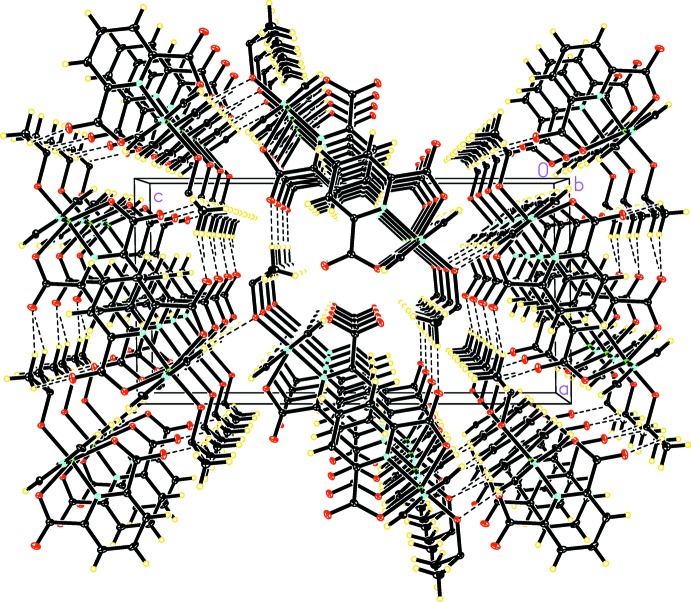
Crystal packing of the title compound, showing hydrogen-bonding inter­actions as dashed lines.

In contrast to the π–π stacking and C—H⋯π inter­actions forming the inter­ior of the double-chains, the exterior of the double-chains is mainly occupied by polar DMSO mol­ecules and carboxyl­ate groups. As a result, a network of C—H⋯O hydrogen bonds exists in the space between the double-chains (Fig. 3 ▸), linking double-chains to form a three dimensional network. Double-chains of mol­ecule *B* are linked by C21*B*—H21*A*⋯O2*B* ^ii^ to form sheets parallel to (001). Double-chains of mol­ecule *A* are linked by C21*A*—H21*E*⋯O2*A* ^i/iv^, C12*A*—H12*A*⋯O1*A* ^i^, C21*A*—H21*D*⋯O4*A* ^iii^, and C22*A*—H22*D*⋯O4*A* ^iii^ hydrogen bonds to form sheets extending along the same direction. Thus, alternating sheets with an *ABAB* pattern can be observed. Two neighboring sheets are connected *via* C11*A*—H11*A*⋯O5*B* and C11*B*—H11*B*⋯O5*A* hydrogen bonds to form a three-dimensional network. The hydrogen-bond lengths and angles are summarized in Table 1 ▸.

In summary, a separation of dissimilar inter­actions can be observed between the non-covalent lipophilic π–π stacking and C—H⋯π inter­actions in the inter­ior of the double-chains and the polar hydrogen bonds in the exterior of the double-chains, further stabilizing the crystal structure.

## Synthesis and crystallization   

Anhydrous NiCO_3_ (0.67 mmol, 79.15 mg), 2,6-pyridine di­carb­oxy­lic acid (0.67 mmol, 111.41 mg), and pyrazine (1.00 mmol, 80.09 mg) were dissolved in 10 ml dimethyl sulfoxide. The resulting mixture was transferred into a stainless steel autoclave which was heated at 373 K for 24 h and cooled to room temperature at a cooling rate of 0.1 K per minute. Green needle-like crystals of the title compound were collected by filtration. Selected IR bands (KBr, cm^−1^): 1640.6 (C=O), 1367.9 (C—O), 950.9 (S=O), 480.6 (bridging pyrazine).

## Refinement   

Crystal data, data collection and structure refinement details are summarized in Table 2 ▸. All H atoms were positioned geometrically (C—H = 0.93/1.00 Å) and allowed to ride with *U*_iso_(H)= 1.2/1.5*U*_eq_(C). Methyl H atoms were allowed to rotate around the corresponding C—C bond. There are two disordered parts, both of which are in mol­ecule *A*. The carboxyl­ate atom O2*A* sits just outside of the mirror plane (occupancy 0.5) and one of the DMSO methyl groups is disordered over two positions in a ratio of 0.54 (2):0.46 (2). The C atom of this group was refined with isotropic displacement parameters.

## Supplementary Material

Crystal structure: contains datablock(s) I. DOI: 10.1107/S2056989016007064/wm5288sup1.cif

Structure factors: contains datablock(s) I. DOI: 10.1107/S2056989016007064/wm5288Isup2.hkl

CCDC reference: 1476677

Additional supporting information:  crystallographic information; 3D view; checkCIF report

## Figures and Tables

**Table 1:** Hydrogen-bond geometry (Å, °)

*D*—H⋯*A*	*D*—H	H⋯*A*	*D*⋯*A*	*D*—H⋯*A*
C11*B*—H11*B*⋯O1*B*	0.95	2.50	3.0442 (13)	117
C11*B*—H11*B*⋯O5*A*	0.95	2.66	3.2871 (18)	124
C11*A*—H11*A*⋯O3*A*	0.95	2.42	3.0252 (14)	121
C11*A*—H11*A*⋯O5*B*	0.95	2.43	3.0462 (17)	122
C12*B*—H12*B*⋯O3*B*	0.95	2.37	2.9978 (13)	123
C12*A*—H12*A*⋯O1*A*	0.95	2.45	3.0221 (14)	119
C12*A*—H12*A*⋯O1*A* ^i^	0.95	2.61	3.2230 (18)	122
C21*B*—H21*A*⋯O2*B* ^ii^	0.98	2.49	3.3321 (19)	144
C21*A*—H21*D*⋯O4*A* ^iii^	0.98	2.47	3.277 (4)	139
C21*A*—H21*E*⋯O2*A* ^i^	0.98	2.27	2.959 (9)	126
C21*A*—H21*E*⋯O2*A* ^iv^	0.98	2.50	3.246 (9)	132
C22*A*—H22*A*⋯O4*A* ^iii^	0.98	2.57	3.377 (4)	140

Symmetry codes: (i) 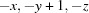; (ii) 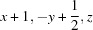; (iii) 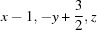; (iv) 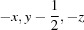.

**Table 2:** Experimental details

Crystal data	
Chemical formula	[Ni(C_7_H_3_NO_4_)(C_4_H_4_N_2_)(C_2_H_6_OS)]
*M*_r_	382.03
Crystal system, space group	Monoclinic, *P*2_1_/*m*
Temperature (K)	100
*a*, *b*, *c* (Å)	10.5631 (7), 7.0296 (4), 20.3710 (13)
β (°)	90.6447 (11)
*V* (Å^3^)	1512.54 (16)
*Z*	4
Radiation type	Mo *K*α
μ (mm^−1^)	1.45
Crystal size (mm)	0.37 × 0.15 × 0.05

Data collection	
Diffractometer	Bruker APEXII DUO CCD
Absorption correction	Analytical based on measured indexed crystal faces; *XPREP* (Bruker, 2014 ▸)
*T*_min_, *T*_max_	0.730, 0.965
No. of measured, independent and observed [*I* > 2σ(*I*)] reflections	56634, 3756, 3549
*R*_int_	0.026
(sin θ/λ)_max_ (Å^−1^)	0.650

Refinement	
*R*[*F* ^2^ > 2σ(*F* ^2^)], *wR*(*F* ^2^), *S*	0.020, 0.055, 1.07
No. of reflections	3756
No. of parameters	256
H-atom treatment	H-atom parameters constrained
Δρ_max_, Δρ_min_ (e Å^−3^)	0.43, −0.31

Computer programs: *APEX2* and *SAINT* (Bruker, 2014 ▸), *SHELXT* (Sheldrick, 2015*a* ▸), *SHELXL2014* (Sheldrick, 2015*b* ▸), *XP* (Bruker, 2014 ▸) and *publCIF* (Westrip, 2010 ▸).

## supplementary crystallographic information

### Crystal data

**Table:**

[Ni(C_7_H_3_NO_4_)(C_4_H_4_N_2_)(C_2_H_6_OS)]	*F*(000) = 784
*M**_r_* = 382.03	*D*_x_ = 1.678 Mg m^−^^3^
Monoclinic, *P*2_1_/*m*	Mo *K*α radiation, λ = 0.71073 Å
*a* = 10.5631 (7) Å	Cell parameters from 9922 reflections
*b* = 7.0296 (4) Å	θ = 2.0–28.0°
*c* = 20.3710 (13) Å	µ = 1.45 mm^−^^1^
β = 90.6447 (11)°	*T* = 100 K
*V* = 1512.54 (16) Å^3^	Needle, green
*Z* = 4	0.37 × 0.15 × 0.05 mm

### Data collection

**Table:**

Bruker APEXII DUO CCD diffractometer	3549 reflections with *I* > 2σ(*I*)
Radiation source: fine-focus sealed tube	*R*_int_ = 0.026
phi and ω scans	θ_max_ = 27.5°, θ_min_ = 1.0°
Absorption correction: analytical based on measured indexed crystal faces; *XPREP* (Bruker, 2014)	*h* = −13→13
*T*_min_ = 0.730, *T*_max_ = 0.965	*k* = −9→9
56634 measured reflections	*l* = −26→26
3756 independent reflections	

### Refinement

**Table:**

Refinement on *F*^2^	Primary atom site location: structure-invariant direct methods
Least-squares matrix: full	Secondary atom site location: difference Fourier map
*R*[*F*^2^ > 2σ(*F*^2^)] = 0.020	Hydrogen site location: inferred from neighbouring sites
*wR*(*F*^2^) = 0.055	H-atom parameters constrained
*S* = 1.07	*w* = 1/[σ^2^(*F*_o_^2^) + (0.0274*P*)^2^ + 1.0377*P*] where *P* = (*F*_o_^2^ + 2*F*_c_^2^)/3
3756 reflections	(Δ/σ)_max_ = 0.001
256 parameters	Δρ_max_ = 0.43 e Å^−^^3^
0 restraints	Δρ_min_ = −0.31 e Å^−^^3^

### Special details

**Table:**

Geometry. All esds (except the esd in the dihedral angle between two l.s. planes) are estimated using the full covariance matrix. The cell esds are taken into account individually in the estimation of esds in distances, angles and torsion angles; correlations between esds in cell parameters are only used when they are defined by crystal symmetry. An approximate (isotropic) treatment of cell esds is used for estimating esds involving l.s. planes.

### Fractional atomic coordinates and isotropic or equivalent isotropic displacement parameters (Å^2^)

**Table:**

	*x*	*y*	*z*	*U*_iso_*/*U*_eq_	Occ. (<1)
Ni1A	0.19703 (2)	0.7500	0.11633 (2)	0.00831 (6)	
Ni1B	0.27491 (2)	0.2500	0.36354 (2)	0.00767 (6)	
S1B	0.54518 (4)	0.2500	0.28209 (2)	0.01208 (9)	
S1A	−0.08847 (4)	0.7500	0.16036 (2)	0.01425 (10)	
O1B	0.10195 (12)	0.2500	0.30920 (6)	0.0113 (2)	
O1A	0.11570 (12)	0.7500	0.02161 (6)	0.0132 (3)	
O2B	−0.10902 (13)	0.2500	0.32425 (7)	0.0233 (3)	
O2A	0.17059 (18)	0.7205 (10)	−0.08458 (9)	0.0253 (15)	0.5
O3B	0.39180 (12)	0.2500	0.44805 (6)	0.0111 (2)	
O3A	0.34526 (12)	0.7500	0.18730 (6)	0.0120 (3)	
O4A	0.55732 (14)	0.7500	0.19630 (8)	0.0263 (4)	
O4B	0.38006 (13)	0.2500	0.55830 (7)	0.0182 (3)	
O5B	0.40109 (12)	0.2500	0.28824 (6)	0.0121 (3)	
O5A	0.05126 (12)	0.7500	0.18052 (6)	0.0133 (3)	
N1B	0.14803 (14)	0.2500	0.43438 (7)	0.0096 (3)	
N1A	0.34951 (14)	0.7500	0.06078 (8)	0.0115 (3)	
N2B	0.27691 (9)	0.55104 (16)	0.36044 (5)	0.0099 (2)	
N2A	0.18744 (9)	0.44903 (16)	0.11862 (5)	0.0106 (2)	
C1A	0.19489 (19)	0.7500	−0.02550 (10)	0.0191 (4)	
C1B	0.33304 (17)	0.2500	0.50290 (9)	0.0116 (3)	
C2B	0.18885 (17)	0.2500	0.49647 (9)	0.0114 (3)	
C2A	0.33379 (18)	0.7500	−0.00413 (9)	0.0145 (4)	
C3B	0.10332 (18)	0.2500	0.54760 (9)	0.0156 (4)	
H3BA	0.1317	0.2500	0.5920	0.019*	
C3A	0.4378 (2)	0.7500	−0.04533 (10)	0.0186 (4)	
H3AA	0.4274	0.7500	−0.0917	0.022*	
C4B	−0.02551 (19)	0.2500	0.53191 (10)	0.0178 (4)	
H4BA	−0.0861	0.2500	0.5660	0.021*	
C4A	0.55810 (19)	0.7500	−0.01630 (11)	0.0206 (4)	
H4AA	0.6310	0.7500	−0.0432	0.025*	
C5B	−0.06604 (18)	0.2500	0.46662 (10)	0.0157 (4)	
H5BA	−0.1537	0.2500	0.4556	0.019*	
C5A	0.57227 (18)	0.7500	0.05169 (11)	0.0183 (4)	
H5AA	0.6540	0.7500	0.0717	0.022*	
C6B	0.02532 (17)	0.2500	0.41808 (9)	0.0114 (3)	
C6A	0.46371 (17)	0.7500	0.08948 (9)	0.0135 (4)	
C7B	0.00208 (17)	0.2500	0.34383 (9)	0.0127 (3)	
C7A	0.45721 (17)	0.7500	0.16448 (9)	0.0141 (4)	
C11B	0.21031 (11)	0.65089 (18)	0.31586 (6)	0.0107 (2)	
H11B	0.1620	0.5849	0.2835	0.013*	
C11A	0.25161 (12)	0.34864 (19)	0.16410 (6)	0.0129 (2)	
H11A	0.2982	0.4144	0.1972	0.015*	
C12B	0.34543 (11)	0.65142 (18)	0.40430 (6)	0.0115 (2)	
H12B	0.3951	0.5856	0.4361	0.014*	
C12A	0.11868 (12)	0.34903 (19)	0.07512 (7)	0.0157 (3)	
H12A	0.0686	0.4149	0.0435	0.019*	
C21B	0.60288 (13)	0.4416 (2)	0.33098 (7)	0.0209 (3)	
H21A	0.6954	0.4467	0.3284	0.031*	
H21B	0.5669	0.5614	0.3148	0.031*	
H21C	0.5781	0.4221	0.3767	0.031*	
C21A	−0.1608 (4)	0.5556 (5)	0.1942 (4)	0.0179 (12)*	0.46 (2)
H21D	−0.2505	0.5546	0.1814	0.027*	0.46 (2)
H21E	−0.1201	0.4393	0.1783	0.027*	0.46 (2)
H21F	−0.1530	0.5616	0.2421	0.027*	0.46 (2)
C22A	−0.1502 (4)	0.5609 (5)	0.2133 (4)	0.0196 (10)*	0.54 (2)
H22A	−0.2413	0.5458	0.2051	0.029*	0.54 (2)
H22B	−0.1068	0.4412	0.2036	0.029*	0.54 (2)
H22C	−0.1356	0.5949	0.2594	0.029*	0.54 (2)

### Atomic displacement parameters (Å^2^)

**Table:**

	*U*^11^	*U*^22^	*U*^33^	*U*^12^	*U*^13^	*U*^23^
Ni1A	0.00710 (11)	0.00803 (11)	0.00981 (11)	0.000	−0.00020 (8)	0.000
Ni1B	0.00712 (11)	0.00684 (11)	0.00903 (11)	0.000	−0.00055 (8)	0.000
S1B	0.0106 (2)	0.0141 (2)	0.0116 (2)	0.000	0.00228 (15)	0.000
S1A	0.0095 (2)	0.0211 (2)	0.0121 (2)	0.000	0.00003 (15)	0.000
O1B	0.0099 (6)	0.0118 (6)	0.0122 (6)	0.000	−0.0019 (5)	0.000
O1A	0.0128 (6)	0.0142 (6)	0.0125 (6)	0.000	−0.0013 (5)	0.000
O2B	0.0098 (6)	0.0403 (9)	0.0198 (7)	0.000	−0.0042 (5)	0.000
O2A	0.0269 (9)	0.038 (5)	0.0115 (7)	−0.0012 (11)	−0.0023 (6)	−0.0008 (10)
O3B	0.0108 (6)	0.0104 (6)	0.0120 (6)	0.000	−0.0013 (5)	0.000
O3A	0.0105 (6)	0.0127 (6)	0.0129 (6)	0.000	−0.0007 (5)	0.000
O4A	0.0115 (7)	0.0433 (10)	0.0239 (8)	0.000	−0.0050 (6)	0.000
O4B	0.0181 (7)	0.0235 (7)	0.0129 (6)	0.000	−0.0057 (5)	0.000
O5B	0.0101 (6)	0.0158 (6)	0.0104 (6)	0.000	−0.0005 (5)	0.000
O5A	0.0079 (6)	0.0187 (7)	0.0133 (6)	0.000	0.0002 (5)	0.000
N1B	0.0100 (7)	0.0073 (7)	0.0116 (7)	0.000	−0.0001 (5)	0.000
N1A	0.0115 (7)	0.0093 (7)	0.0135 (7)	0.000	0.0014 (6)	0.000
N2B	0.0089 (5)	0.0089 (5)	0.0118 (5)	0.0000 (4)	0.0020 (4)	−0.0003 (4)
N2A	0.0091 (5)	0.0101 (5)	0.0128 (5)	0.0004 (4)	0.0015 (4)	−0.0001 (4)
C1A	0.0179 (9)	0.0238 (10)	0.0156 (9)	0.000	−0.0018 (7)	0.000
C1B	0.0131 (8)	0.0067 (8)	0.0151 (9)	0.000	−0.0019 (7)	0.000
C2B	0.0137 (8)	0.0083 (8)	0.0120 (8)	0.000	−0.0008 (7)	0.000
C2A	0.0166 (9)	0.0123 (8)	0.0145 (9)	0.000	0.0023 (7)	0.000
C3B	0.0195 (9)	0.0161 (9)	0.0113 (8)	0.000	0.0016 (7)	0.000
C3A	0.0233 (10)	0.0171 (9)	0.0155 (9)	0.000	0.0057 (8)	0.000
C4B	0.0169 (9)	0.0194 (10)	0.0172 (9)	0.000	0.0080 (7)	0.000
C4A	0.0169 (9)	0.0183 (10)	0.0268 (11)	0.000	0.0123 (8)	0.000
C5B	0.0116 (8)	0.0160 (9)	0.0196 (9)	0.000	0.0018 (7)	0.000
C5A	0.0113 (9)	0.0159 (9)	0.0278 (11)	0.000	0.0037 (8)	0.000
C6B	0.0102 (8)	0.0096 (8)	0.0144 (8)	0.000	−0.0002 (7)	0.000
C6A	0.0118 (8)	0.0098 (8)	0.0190 (9)	0.000	0.0010 (7)	0.000
C7B	0.0119 (8)	0.0114 (8)	0.0149 (9)	0.000	−0.0015 (7)	0.000
C7A	0.0111 (8)	0.0123 (9)	0.0188 (9)	0.000	−0.0011 (7)	0.000
C11B	0.0119 (5)	0.0108 (6)	0.0094 (5)	−0.0005 (5)	0.0014 (4)	−0.0009 (5)
C11A	0.0169 (6)	0.0122 (6)	0.0095 (5)	−0.0007 (5)	0.0000 (4)	−0.0009 (5)
C12B	0.0095 (5)	0.0106 (6)	0.0144 (6)	0.0004 (5)	−0.0009 (4)	0.0007 (5)
C12A	0.0121 (6)	0.0122 (7)	0.0226 (7)	0.0006 (5)	−0.0067 (5)	0.0009 (5)
C21B	0.0136 (6)	0.0194 (7)	0.0295 (7)	−0.0040 (5)	0.0011 (5)	−0.0080 (6)

### Geometric parameters (Å, º)

**Table:**

Ni1A—N1A	1.9788 (15)	C1A—O2A^i^	1.245 (3)
Ni1A—O5A	2.0313 (13)	C1A—C2A	1.526 (3)
Ni1A—O1A	2.1032 (13)	C1B—C2B	1.527 (2)
Ni1A—N2A^i^	2.1186 (11)	C2B—C3B	1.386 (3)
Ni1A—N2A	2.1186 (11)	C2A—C3A	1.390 (3)
Ni1A—O3A	2.1191 (13)	C3B—C4B	1.394 (3)
Ni1B—N1B	1.9804 (15)	C3B—H3BA	0.9500
Ni1B—O5B	2.0434 (13)	C3A—C4A	1.395 (3)
Ni1B—O3B	2.1073 (13)	C3A—H3AA	0.9500
Ni1B—N2B	2.1172 (11)	C4B—C5B	1.393 (3)
Ni1B—N2B^ii^	2.1173 (11)	C4B—H4BA	0.9500
Ni1B—O1B	2.1255 (12)	C4A—C5A	1.391 (3)
S1B—O5B	1.5286 (13)	C4A—H4AA	0.9500
S1B—C21B^ii^	1.7786 (14)	C5B—C6B	1.389 (3)
S1B—C21B	1.7786 (14)	C5B—H5BA	0.9500
S1A—O5A	1.5276 (13)	C5A—C6A	1.388 (3)
S1A—C21A	1.713 (4)	C5A—H5AA	0.9500
S1A—C21A^i^	1.713 (4)	C6B—C7B	1.530 (3)
S1A—C22A	1.836 (4)	C6A—C7A	1.530 (3)
S1A—C22A^i^	1.836 (4)	C11B—C11B^i^	1.393 (3)
O1B—C7B	1.276 (2)	C11B—H11B	0.9500
O1A—C1A	1.280 (2)	C11A—C11A^ii^	1.387 (3)
O2B—C7B	1.235 (2)	C11A—H11A	0.9500
O2A—O2A^i^	0.414 (15)	C12B—C12B^i^	1.386 (3)
O2A—C1A	1.245 (3)	C12B—H12B	0.9500
O3B—C1B	1.284 (2)	C12A—C12A^ii^	1.392 (3)
O3A—C7A	1.276 (2)	C12A—H12A	0.9500
O4A—C7A	1.234 (2)	C21B—H21A	0.9800
O4B—C1B	1.228 (2)	C21B—H21B	0.9800
N1B—C2B	1.332 (2)	C21B—H21C	0.9800
N1B—C6B	1.334 (2)	C21A—H21D	0.9800
N1A—C2A	1.331 (2)	C21A—H21E	0.9800
N1A—C6A	1.335 (2)	C21A—H21F	0.9800
N2B—C11B	1.3408 (16)	C22A—H22A	0.9800
N2B—C12B	1.3438 (16)	C22A—H22B	0.9800
N2A—C12A	1.3388 (17)	C22A—H22C	0.9800
N2A—C11A	1.3423 (16)		
			
N1A—Ni1A—O5A	174.81 (6)	N1B—C2B—C3B	120.45 (17)
N1A—Ni1A—O1A	78.58 (6)	N1B—C2B—C1B	113.16 (15)
O5A—Ni1A—O1A	106.61 (5)	C3B—C2B—C1B	126.39 (17)
N1A—Ni1A—N2A^i^	92.98 (3)	N1A—C2A—C3A	120.61 (18)
O5A—Ni1A—N2A^i^	87.08 (3)	N1A—C2A—C1A	113.10 (16)
O1A—Ni1A—N2A^i^	90.06 (3)	C3A—C2A—C1A	126.30 (18)
N1A—Ni1A—N2A	92.98 (3)	C2B—C3B—C4B	118.06 (17)
O5A—Ni1A—N2A	87.08 (3)	C2B—C3B—H3BA	121.0
O1A—Ni1A—N2A	90.06 (3)	C4B—C3B—H3BA	121.0
N2A^i^—Ni1A—N2A	173.95 (6)	C2A—C3A—C4A	117.80 (18)
N1A—Ni1A—O3A	77.89 (6)	C2A—C3A—H3AA	121.1
O5A—Ni1A—O3A	96.92 (5)	C4A—C3A—H3AA	121.1
O1A—Ni1A—O3A	156.48 (5)	C5B—C4B—C3B	120.50 (17)
N2A^i^—Ni1A—O3A	91.15 (3)	C5B—C4B—H4BA	119.8
N2A—Ni1A—O3A	91.15 (3)	C3B—C4B—H4BA	119.8
N1B—Ni1B—O5B	178.13 (6)	C5A—C4A—C3A	120.61 (18)
N1B—Ni1B—O3B	78.45 (6)	C5A—C4A—H4AA	119.7
O5B—Ni1B—O3B	103.42 (5)	C3A—C4A—H4AA	119.7
N1B—Ni1B—N2B	91.65 (3)	C6B—C5B—C4B	118.12 (17)
O5B—Ni1B—N2B	88.32 (3)	C6B—C5B—H5BA	120.9
O3B—Ni1B—N2B	91.06 (3)	C4B—C5B—H5BA	120.9
N1B—Ni1B—N2B^ii^	91.65 (3)	C6A—C5A—C4A	118.14 (18)
O5B—Ni1B—N2B^ii^	88.32 (3)	C6A—C5A—H5AA	120.9
O3B—Ni1B—N2B^ii^	91.06 (3)	C4A—C5A—H5AA	120.9
N2B—Ni1B—N2B^ii^	176.38 (6)	N1B—C6B—C5B	120.23 (17)
N1B—Ni1B—O1B	78.15 (6)	N1B—C6B—C7B	112.99 (15)
O5B—Ni1B—O1B	99.97 (5)	C5B—C6B—C7B	126.78 (16)
O3B—Ni1B—O1B	156.61 (5)	N1A—C6A—C5A	120.34 (18)
N2B—Ni1B—O1B	89.61 (3)	N1A—C6A—C7A	112.76 (16)
N2B^ii^—Ni1B—O1B	89.61 (3)	C5A—C6A—C7A	126.89 (17)
O5B—S1B—C21B^ii^	106.82 (6)	O2B—C7B—O1B	127.59 (18)
O5B—S1B—C21B	106.82 (6)	O2B—C7B—C6B	117.43 (16)
C21B^ii^—S1B—C21B	98.42 (11)	O1B—C7B—C6B	114.98 (15)
O5A—S1A—C21A	109.0 (2)	O4A—C7A—O3A	126.94 (18)
O5A—S1A—C21A^i^	109.0 (2)	O4A—C7A—C6A	118.47 (17)
C21A—S1A—C21A^i^	105.8 (3)	O3A—C7A—C6A	114.59 (16)
O5A—S1A—C22A	101.00 (18)	N2B—C11B—C11B^i^	121.57 (7)
O5A—S1A—C22A^i^	101.00 (18)	N2B—C11B—H11B	119.2
C22A—S1A—C22A^i^	92.8 (3)	C11B^i^—C11B—H11B	119.2
C7B—O1B—Ni1B	115.04 (11)	N2A—C11A—C11A^ii^	121.72 (7)
C1A—O1A—Ni1A	115.10 (12)	N2A—C11A—H11A	119.1
O2A^i^—O2A—C1A	80.4 (3)	C11A^ii^—C11A—H11A	119.1
C1B—O3B—Ni1B	115.23 (11)	N2B—C12B—C12B^i^	121.67 (7)
C7A—O3A—Ni1A	115.61 (12)	N2B—C12B—H12B	119.2
S1B—O5B—Ni1B	136.06 (8)	C12B^i^—C12B—H12B	119.2
S1A—O5A—Ni1A	124.34 (8)	N2A—C12A—C12A^ii^	121.68 (7)
C2B—N1B—C6B	122.64 (16)	N2A—C12A—H12A	119.2
C2B—N1B—Ni1B	118.53 (12)	C12A^ii^—C12A—H12A	119.2
C6B—N1B—Ni1B	118.83 (12)	S1B—C21B—H21A	109.5
C2A—N1A—C6A	122.50 (16)	S1B—C21B—H21B	109.5
C2A—N1A—Ni1A	118.36 (13)	H21A—C21B—H21B	109.5
C6A—N1A—Ni1A	119.14 (13)	S1B—C21B—H21C	109.5
C11B—N2B—C12B	116.75 (11)	H21A—C21B—H21C	109.5
C11B—N2B—Ni1B	122.56 (8)	H21B—C21B—H21C	109.5
C12B—N2B—Ni1B	120.69 (8)	S1A—C21A—H21D	109.5
C12A—N2A—C11A	116.53 (12)	S1A—C21A—H21E	109.5
C12A—N2A—Ni1A	122.38 (9)	H21D—C21A—H21E	109.5
C11A—N2A—Ni1A	121.09 (9)	S1A—C21A—H21F	109.5
O2A—C1A—O2A^i^	19.2 (7)	H21D—C21A—H21F	109.5
O2A—C1A—O1A	126.5 (2)	H21E—C21A—H21F	109.5
O2A^i^—C1A—O1A	126.5 (2)	S1A—C22A—H22A	109.5
O2A—C1A—C2A	117.57 (19)	S1A—C22A—H22B	109.5
O2A^i^—C1A—C2A	117.57 (19)	H22A—C22A—H22B	109.5
O1A—C1A—C2A	114.86 (17)	S1A—C22A—H22C	109.5
O4B—C1B—O3B	127.24 (17)	H22A—C22A—H22C	109.5
O4B—C1B—C2B	118.13 (16)	H22B—C22A—H22C	109.5
O3B—C1B—C2B	114.63 (15)		

Symmetry codes: (i) *x*, −*y*+3/2, *z*; (ii) *x*, −*y*+1/2, *z*.

### Hydrogen-bond geometry (Å, º)

**Table:**

*D*—H···*A*	*D*—H	H···*A*	*D*···*A*	*D*—H···*A*
C11*B*—H11*B*···O1*B*	0.95	2.50	3.0442 (13)	117
C11*B*—H11*B*···O5*A*	0.95	2.66	3.2871 (18)	124
C11*A*—H11*A*···O3*A*	0.95	2.42	3.0252 (14)	121
C11*A*—H11*A*···O5*B*	0.95	2.43	3.0462 (17)	122
C12*B*—H12*B*···O3*B*	0.95	2.37	2.9978 (13)	123
C12*A*—H12*A*···O1*A*	0.95	2.45	3.0221 (14)	119
C12*A*—H12*A*···O1*A*^iii^	0.95	2.61	3.2230 (18)	122
C21*B*—H21*A*···O2*B*^iv^	0.98	2.49	3.3321 (19)	144
C21*A*—H21*D*···O4*A*^v^	0.98	2.47	3.277 (4)	139
C21*A*—H21*E*···O2*A*^iii^	0.98	2.27	2.959 (9)	126
C21*A*—H21*E*···O2*A*^vi^	0.98	2.50	3.246 (9)	132
C22*A*—H22*A*···O4*A*^v^	0.98	2.57	3.377 (4)	140

Symmetry codes: (iii) −*x*, −*y*+1, −*z*; (iv) *x*+1, −*y*+1/2, *z*; (v) *x*−1, −*y*+3/2, *z*; (vi) −*x*, *y*−1/2, −*z*.

## References

[bb1] Bruker (2014). *APEX2*, *SAINT*, *XPREP* and *XP*. Bruker Inc., Madison, Wisconsin, USA.

[bb2] Felloni, M., Blake, A. J., Hubberstey, P., Teat, S. J., Wilson, C. & Schröder, M. (2010). *CrystEngComm*, **12**, 1576–1589.

[bb3] Hohenstein, E. G. & Sherrill, C. D. (2009). *J. Phys. Chem. A*, **113**, 878–886. 10.1021/jp809062x 19132847

[bb4] Huber, R. G., Margreiter, M. A., Fuchs, J. E., von Grafenstein, S., Tautermann, C. S., Liedl, K. R. & Fox, T. (2014). *J. Chem. Inf. Model.* **54**, 1371–1379. 10.1021/ci500183u PMC4037317 24773380

[bb5] Liu, C., Čižmár, E., Park, J. H., Abboud, K. A., Meisel, M. W. & Talham, D. R. (2011). *Polyhedron*, **30**, 1420–1424.

[bb6] Mishra, B. K. & Sathyamurthy, N. (2005). *J. Phys. Chem. A*, **109**, 6–8. 10.1021/jp045218c 16839083

[bb7] Nawrot, I., Machura, B. & Kruszynski, R. (2015). *CrystEngComm*, **17**, 830–845.

[bb8] Sheldrick, G. M. (2015*a*). *Acta Cryst.* A**71**, 3–8.

[bb9] Sheldrick, G. M. (2015*b*). *Acta Cryst.* C**71**, 3–8.

[bb10] Tiekink, E. R. T. & Zuckerman-Schpector, J. (2012). Editors. *Importance of Pi-interaction in Crystal Engineering: Frontiers in Crystal Engineering*, 2nd ed., pp. 111–112. London: Wiley.

[bb11] Westrip, S. P. (2010). *J. Appl. Cryst.* **43**, 920–925.

[bb12] Zheng, Y. Q., Sun, J. & Lin, J. L. (2000). *Z. Anorg. Allg. Chem.* **626**, 1501–1504.

